# Reaction-Type-Dependent Behavior of Redox-Hopping in MOFs—Does Charge Transport Have a Preferred Direction?

**DOI:** 10.1021/acs.jpclett.4c01674

**Published:** 2024-11-21

**Authors:** Minliang Yan, Zaya Bowman, Zachary J. Knepp, Aiden Peterson, Lisa A. Fredin, Amanda J. Morris

**Affiliations:** † Macromolecules Innovation Institute, Virginia Polytechnic Institute and State University, Blacksburg, Virginia 24061, United States; ‡ Department of Chemical Engineering and Department of Chemistry, Virginia Polytechnic Institute and State University, Blacksburg, Virginia 24061, United States; § Department of Chemistry, Lehigh University, Bethlehem, Pennsylvania 18015, United States; ∥ Department of Chemistry, Virginia Polytechnic Institute and State University, Blacksburg, Virginia 24061, United States

## Abstract

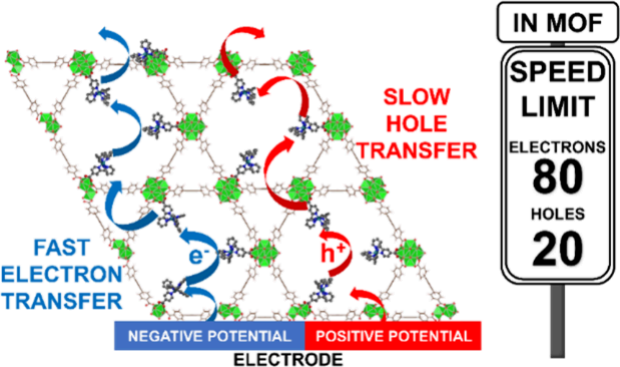

Redox hopping is the primary method of electron transport through redox-active metal–organic frameworks (MOFs). While redox hopping adequately supports the electrocatalytic application of MOFs, the fundamental understandings guiding the design of redox hopping MOFs remain nascent. In this study, we probe the rate of electron and hole transport through a singular MOF scaffold to determine whether the properties of the MOF promote the transport of one carrier over the other. A redox center, [Ru^II^(bpy)_2_(bpy-COOH)]^2+^, where bpy = 2,2′-bipyridine and bpy-COOH = 4-carboxy-2,2′-bipyridine, was anchored within NU-1000. The electron hopping coefficients (*D*_*e*_) and ion diffusion coefficients (*D*_*i*_) were calculated via chronoamperometry and application of the Scholz model. We found that electrons transport more rapidly than holes in the studied MOF. Interestingly, the correlation between *D*_*e*_ and self-exchange rate built in previous research predicted reversely. The contradicting result indicates that spacing between the molecular moieties involved in a particular hopping process dominates the response.

Metal–organic frameworks (MOFs), a class of coordination polymers, are a family of permanently porous materials made through the coordination of organic linkers and metal nodes. MOFs have numerous unique properties: periodic and tunable molecular-scale porosity, high specific surface area, robust structure, variety of topology, geometry, functionality, etc.^1^ With these properties, MOFs have shown promise in applications including but not limited to gas separation, gas storage, drug delivery, catalysis, and energy storage. In recent years, the electrochemical and photoelectrochemical application of MOFs has garnered interest, including thermoelectrics, solar cells, and electrochemical catalysts, which all require conductivity.^2^

Several strategies for imparting conductivity to MOFs can be summarized by two transport mechanisms: band-like transport and hopping transport.^2,3^ Three subtypes of band-like transport are the through-bond, extended-conjugation, and through-space pathways. The through-bond path relies on the orbital overlap between the metal nodes and the functional groups on the organic linker to build the 1-D path for electron transport. The extended conjugation includes the metal node and whole linker, both functional groups and the organic backbone, to form a 2-D delocalized network.^4^ MOF films capable of these two mechanisms have relatively high conductivity. However, to realize conductivity, careful selection of node-linker pairs is required to match the ligand-node orbital energy levels.^2,3^ A through-space pathway is generally observed for the 2D MOFs, which feature closely spaced π–π stacks of conjugated organic linkers. The observed conductivity in such scaffolds depends heavily on the interlayer distance and stacking pattern; consequently, careful design is also required.^2^

Hopping transport is based on electron hopping between spatially separated redox active moieties.^5−7^ In contrast to the strictly matched node-linker pair in the conjugation pathway and the well-designed stacking in the through-space pathway, redox hopping in MOFs can be realized in various ways: redox centers can be metal nodes,^8−13^ organic linkers,^13−23^ guest molecules,^24−28^ etc. Moreover, postsynthetic methods can easily functionalize or anchor the redox centers onto MOFs (e.g., solvent-assistant ligand incorporation (SALI)).^29−32^ Therefore, a simple design and experimental method can incorporate redox-hopping capability into almost any MOF. Even though the conductivity of redox-hopping MOFs is relatively low (1.2 × 10^–7^ S/cm compared to 1,580 S/cm for redox-hopping and band-like transport, respectively),^21,33^ such a mechanism can be exploited in electrocatalytic frameworks, where catalysis is likely to be rate-limiting.

In the previous research on redox-hopping MOFs, a parameter termed the apparent diffusion coefficient (*D*_*app*_) derived from chronoamperometry (CA) via the Cottrell equation was widely used to describe the overall redox-hopping process in MOFs.^7,13,29,32,34−36^ The *D*_*app*_ includes contributions from two interlocked processes: electron hopping between redox centers (also known as self-exchange) and counterion diffusion to neutralize the resultant charge. The Scholz model was recently applied to MOFs to separate these processes into independent parameters, the electron hopping coefficient (*D*_*e*_) and ion diffusion coefficient (*D*_*i*_).^3,4^ The model assumes that the reaction starts at the three-phase junction (electrode/crystallite/electrolyte) and propagates into the crystal. Electron hopping is assumed to be perpendicular to the electrode surface, while the ion diffusion is parallel.^37^ From the previous literature, a few essential properties of redox hopping in MOFs are uncovered: (1) *D*_*e*_ depends on the self-exchange rate between incorporated redox centers. (2) *D*_*i*_ depends on the size of the counterion, generally larger for more compact counterions (3) *D*_*e*_ is generally several orders of magnitude higher than *D*_*i*_, meaning ion diffusion is the limiting process in redox hopping.^2−4^

In this study, we prepared NU-1000 particles with bis(2,2′-bipyridine)(4-carboxy-2,2′-bipyridine) ruthenium(II) ([Ru^II^(bpy)_2_(bpy-COOH)]^2+^) redox centers anchored onto the nodes (Ru-NU-1000) and electrophorectically deposited the Ru-NU-1000 particles onto FTO slides. Considering the nature of the redox center, [Ru^II^(bpy)_2_(bpy-COOH)]^2+^, charge transfer can occur in two directions: when an oxidative potential is applied, the electrons will flow out from the MOF through the highest occupied molecular orbitals (HOMOs) which are filled Ru t_2g_ orbitals; when a reductive potential is applied, the electrons will flow into the MOF to the lowest unoccupied molecular orbitals (LUMOs), which are π* orbitals of the bpy ligands (Figure 1).

**Figure 1:**
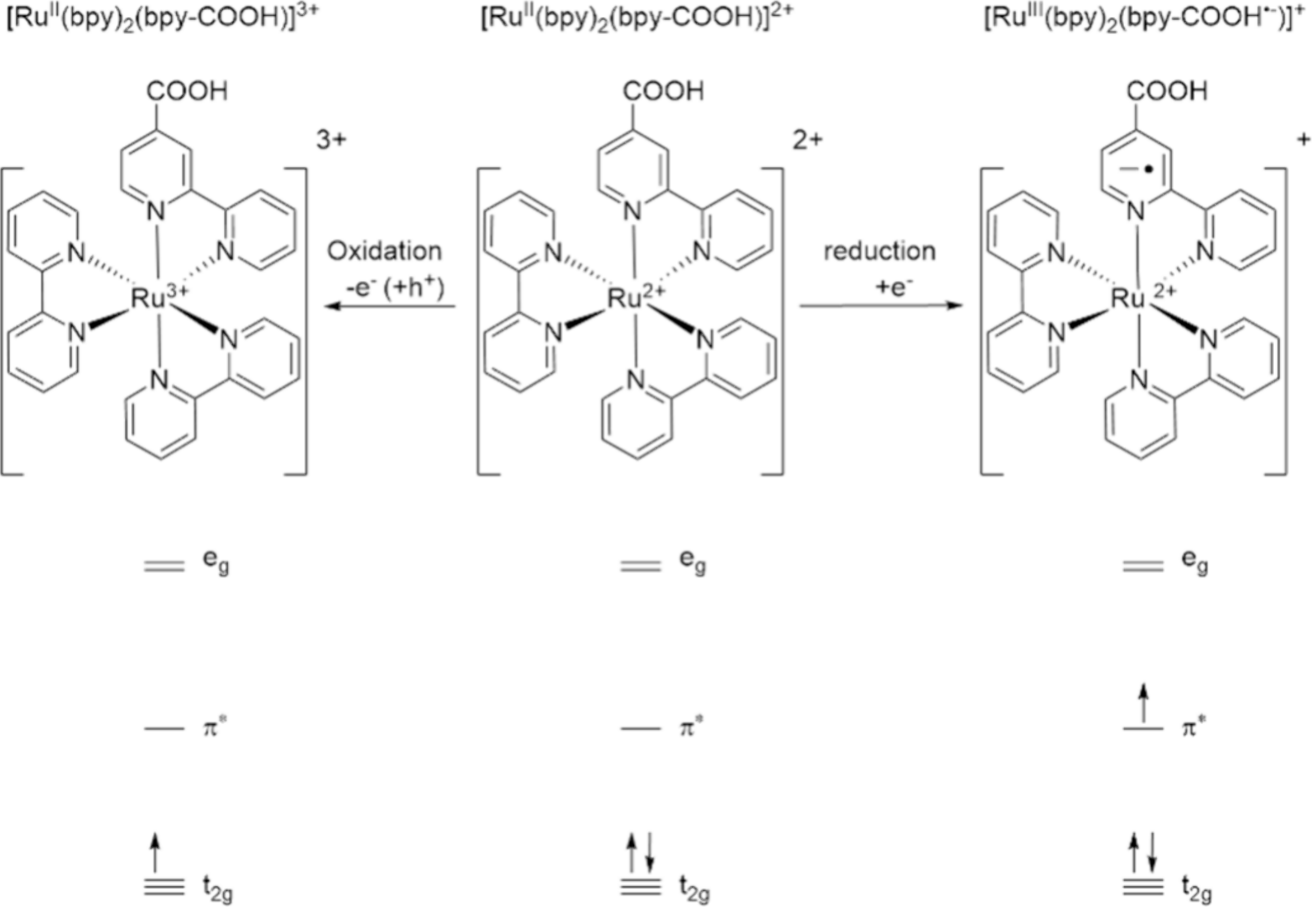
Molecular structures and simplified molecular orbital diagrams of [Ru^II^(bpy)_2_(bpy-COOH^•–^)]^+^ (left), [Ru^II^(bpy)_2_(bpy-COOH)]^2+^ (middle), and [Ru^III^(bpy)_2_(bpy-COOH)]^3+^ (right).

These two pathways can be considered the molecular equivalent to charge transfer in traditional semiconductors: in n-type semiconductors, the charge transfer is dominated by the transfer through the conduction band, while in p-type semiconductors, it is dominantly carried by the valence band. It is well-known that the character of the semiconductor—n-type or p-type—dictates its optimal application in either reductive or oxidative photoelectrocatalytic schemes. From this perspective, we wanted to investigate the potential for a preferred direction of charge transport in redox-hopping MOFs. Herein, we quantified *D*_*e*_ and *D*_*i*_ at the appropriate applied potentials for reduction and oxidation of Ru-NU-1000 via CA and application of the Scholz model.^37^ The results indicated that reductive transport was faster, exhibiting a higher *D*_*e*_ and *D*_*i*_. The role of self-exchange rates, site-to-site hopping distance, steric crowding of the transport channel, and ion pairing are discussed. The results provide clear design rules for optimization in future electrocatalytic MOF applications.

NU-1000 was prepared via previously published procedures.^38^ Structural confirmation was provided via powder X-ray diffraction, scanning electron microscopy, and surface area measurements (see Figure 2 and Supporting Information). A modified SALI procedure was employed to incorporate [Ru^II^(bpy)_2_(bpy-COOH)]^2+^. Briefly, the pristine MOF was heated with the [Ru^II^(bpy)_2_(bpy-COOH)](PF_6_)_2_ in DMF with continuous stirring. The concentration of [Ru^II^(bpy)_2_(bpy-COOH)]^2+^ in Ru-NU-1000 was confirmed and quantified via ^1^H NMR on the acid-digested MOF sample (Figure S5). Immobilization of the Ru-NU-1000 onto the conductive substrate, in this case fluorine-doped tin oxide (FTO), was carried out via electrophoretic deposition (EPD). Scanning electron micrographs confirm the near particle-thick film of isolated crystals, so particle-to-particle hopping contributions to the electrochemical response will be minimized (Figure 2).

**Figure 2:**
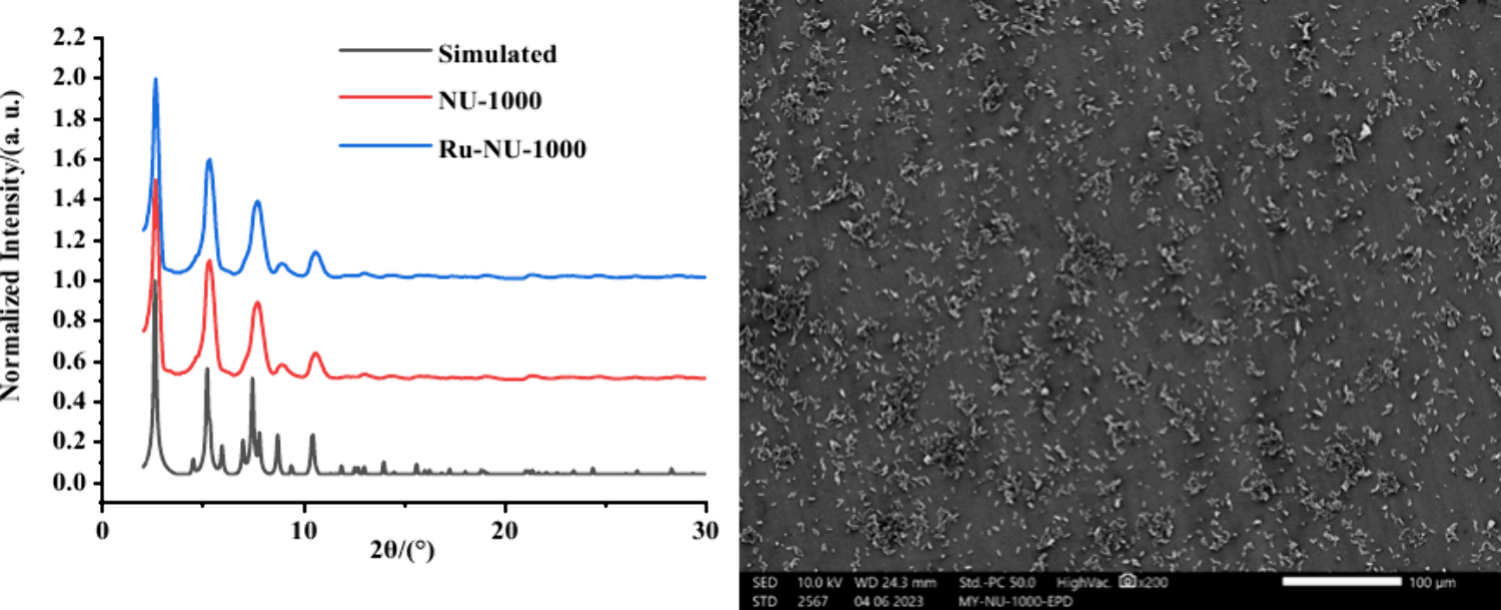
(Left) PXRD pattern of NU-1000 (red), Ru-NU-1000 (blue), and simulated PXRD pattern of NU-1000 (black). (Right) The SEM image of Ru-NU-1000 film prepared by EPD on the FTO slide shows a near particle-thick film distribution.

The electrochemical response of Ru-NU-1000 was investigated by differential pulse voltammetry (DPV) in CH_3_CN with a TBAPF_6_ supporting electrolyte. A DPV scan from an open circuit to 2.0 V vs Ag/AgNO_3_ revealed a single redox event (Figure 3a). Comparison to [Ru^II^(bpy)_2_(bpy-COOH)](PF_6_)_2_ in homogeneous solution (Figure S6) indicated that the response is associated with the anchored redox-active Ru compound. Comparison of the DPV responses of Ru-NU-1000 and pristine NU-1000 (Figure S7b) clearly demonstrated that the peak from the oxidation of NU-1000s pyrene-based linker is presented as a bump at ∼1.6 V, in contrast to the quasi-reversible Ru^2+/3+^ features at ∼1.0 V. The two species are sufficiently separated in oxidation potential, such that the linkers are unlikely to be oxidized under the electrochemical potential step conditions used. Therefore, the peak in the Figure 3a is from the pair of Ru^2+/3+^. The scan-rate-dependent CV showed that the half-wave potential (*E*_1/2_) is ∼1.0 V vs Ag/AgNO_3_ (Figure S7c) at all sweep rates, and the plot of peak current vs scan rate (Figure S7d) showed great linearity, which indicated that the oxidation is diffusion-controlled.

**Figure 3:**
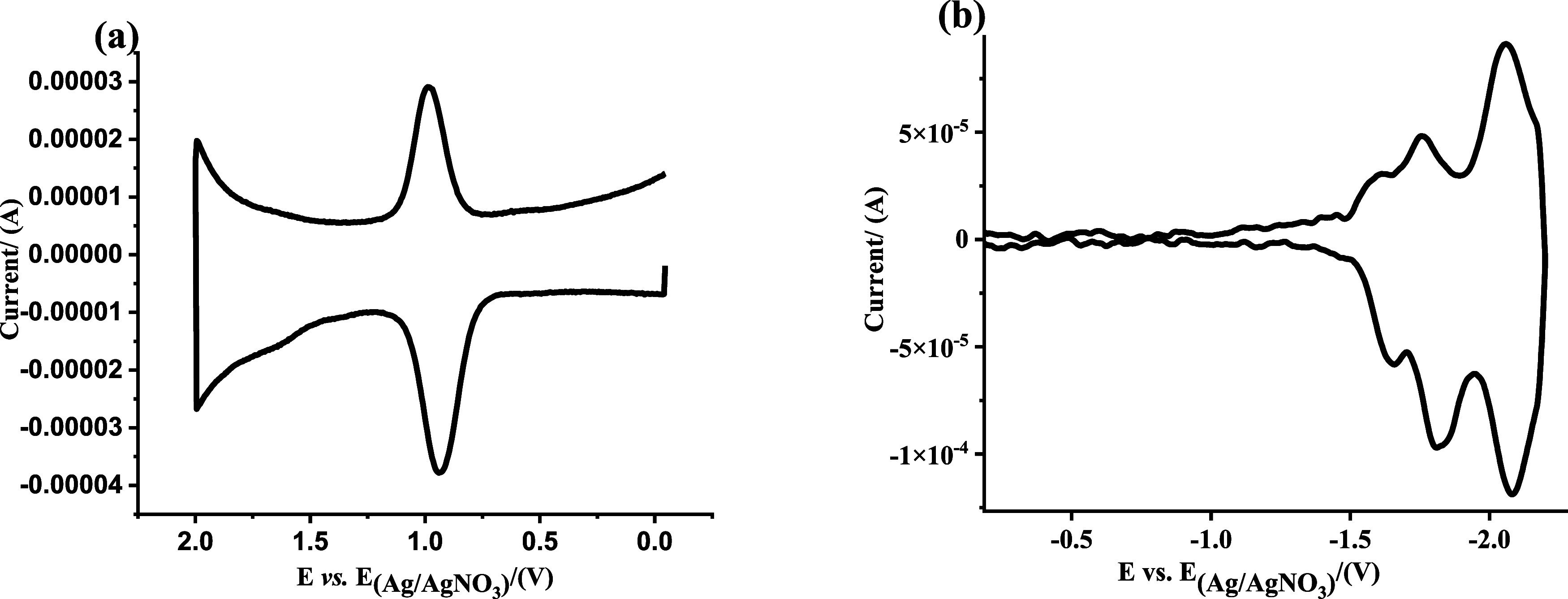
DPV plot of Ru-NU-1000 (a) oxidation and (b) reduction side, in a 0.1 M TBAPF_6_ in acetonitrile solution. Period = 30 ms, width = 50 ms, height = 50 mV, increment = 5 mV.

The DPV scan from open circuit to −2.2 V vs Ag/AgNO_3_ displays three redox waves with *E*_1/2_ of −1.63 V, −1.78 V, and −2.07 V (Figure 3b). Again, comparison to [Ru^II^(bpy)_2_(bpy-COOH)](PF_6_)_2_ compound in homogeneous solution (Figure S8) indicated that the events were associated with the anchored redox-active compound. Previous studies enabled the assignment of these peaks to subsequent reductions of the three bpy ligands.^39,40^ Considering the electron-withdrawing nature of the carboxylic acid group, the reduction of the bpy-COOH ligand should occur at a more positive potential than the other two bpy ligands. Therefore, the reduction signal at −1.63 V was assigned to the bpy-COOH ligand, followed by the remaining two pristine bpy ligands.

Chronoamperometry was performed to quantify the diffusion coefficients for redox hopping. In line with Cottrell analysis, an excitation pulse was conducted from an area of open circuit to beyond the redox wave of interest. The open circuit potential was held for 1 h before switching the excitation potential. In the case of oxidation, the 1.2 V vs Ag/AgNO_3_, at which the oxidation peak of Ru ends, was applied. The difference between the *E*_1/2_ and the excitation pulse ensures that the current measured will be in the diffusion-limited regime. For the reduction, an excitation voltage of −1.65 V was applied. The difference between the excitation pulse and *E*_1/2_ was necessarily smaller due to the potential overlap with the second bpy reduction wave.

The resultant current–voltage plots display the characteristic exponential decay expected for diffusive transport as predicted by the Cottrell model, eq 1.
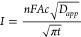
1where *i* is the current, *n* is the number of electrons transferred in the redox reaction, *F* is the Faraday constant, *A* is the area of the planar electrode, *c* is the initial concentration of the redox active species, *D*_*app*_ is the apparent diffusion coefficient of the redox active species, and *t* is the time.

While the *D*_*app*_ is widely reported for redox-hopping MOFs to measure the electron transport rate, we have previously shown that applying a more extended Scholz model is relevant and applicable to MOFs.^3,4,6^ The Scholz model enables the separation of electron and ion transport contributions to *D*_*app*_ by quantifying *D*_*e*_ (electron diffusion) and *D*_*i*_ (ion diffusion). The Scholz model accounts for the electrochemical conversion of molecules at the surface of the MOF crystallites, defined as Stage A in the model, and bulk diffusion, defined as Stage B. To distinguish stage A and stage B, the current–time (*i vs* t) responses (Figure 4a,b) were transformed into  vs  plots (Figure 4c,d). The maximum value of  was denoted as the reference time (*t*_*ref*_) and signified the transition between stages A and B.

**Figure 4:**
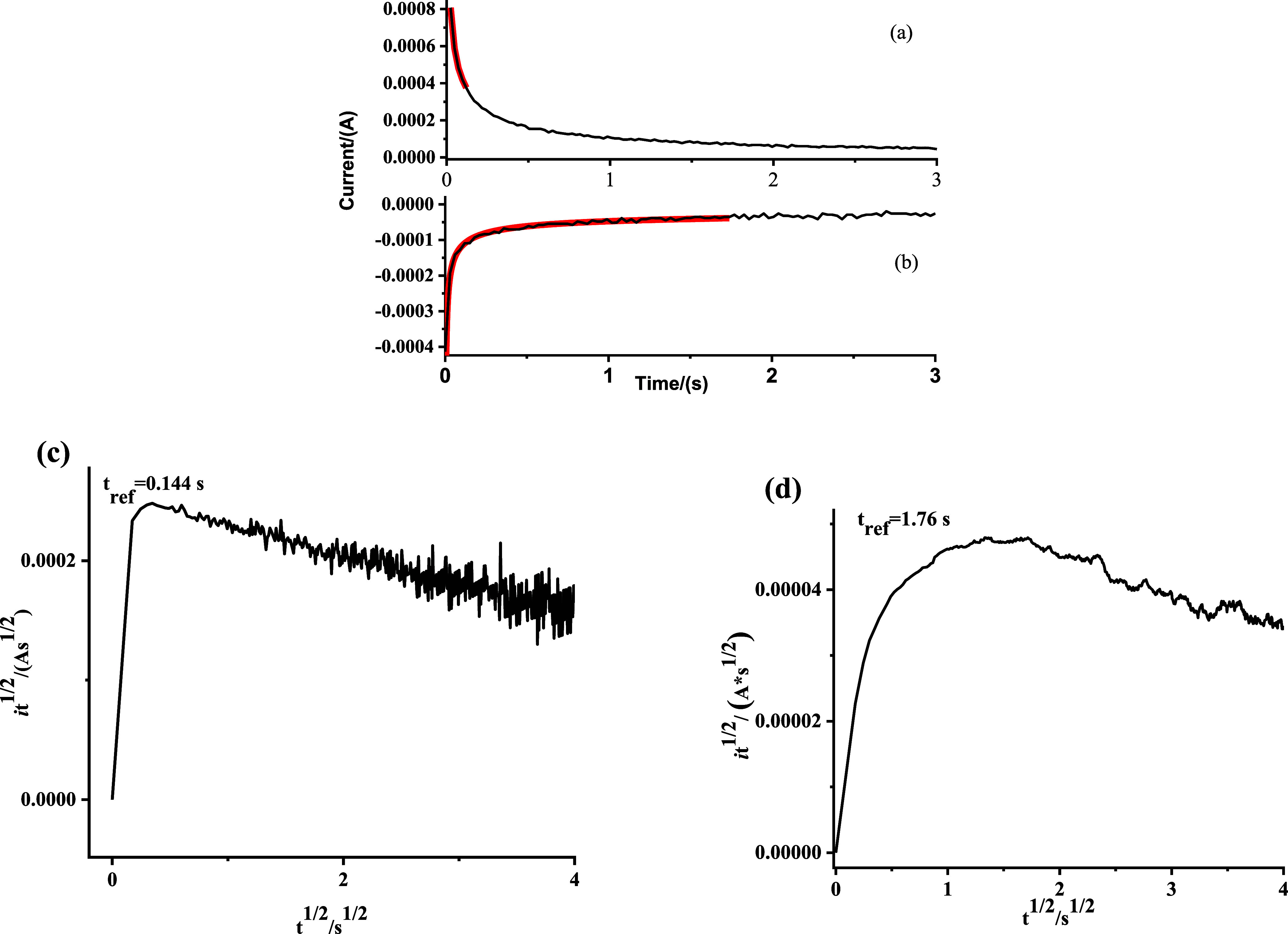
(a,b) Chronoamperograms (black line) and the Stage A Scholz model fits (red line) for (a) reduction and (b) oxidation of Ru-NU-1000. (c,d) The  vs  Scholz plots for reduction (c) and oxidation (d) used to determine *t*_*ref*_.

For stage A, the current–time relationship should follow the eq 2,
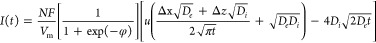
2where *N* is the number of particles in the MOF film, *F* is Faraday’s constant, *V*_m_ is the molar volume of NU-1000, *u* is the length of the three-phase junction (the parameter of particle-electrode interface), *D*_*e*_ is the electron hopping coefficient, *D*_*i*_ is the ion diffusion coefficient, Δ*x*_0_ is the ion hopping distance, and Δ*z*_0_ is the electron hopping distance. φ is defined as
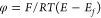
where *F* is Faraday’s constant, *R* is the gas constant, *T* is the temperature, *E* is the applied excitation potential in chronoamperometry, and *E*_*f*_ is the formal potential of the redox pair. All the parameters can be calculated, derived, or determined based on the SEM image, chronoamperometry, and structure of MOF^4^ (see the Supporting Information).

By fitting eq 2 to the chronoamperometry responses before *t*_*ref*_ (Figure 4a,b), *D*_*e*_ and *D*_*i*_ in stage A were calculated. The calculated *D*_*e*_ and *D*_*i*_ are listed in Table 1, and fitting is visualized in Figure 5.

**Figure 5:**
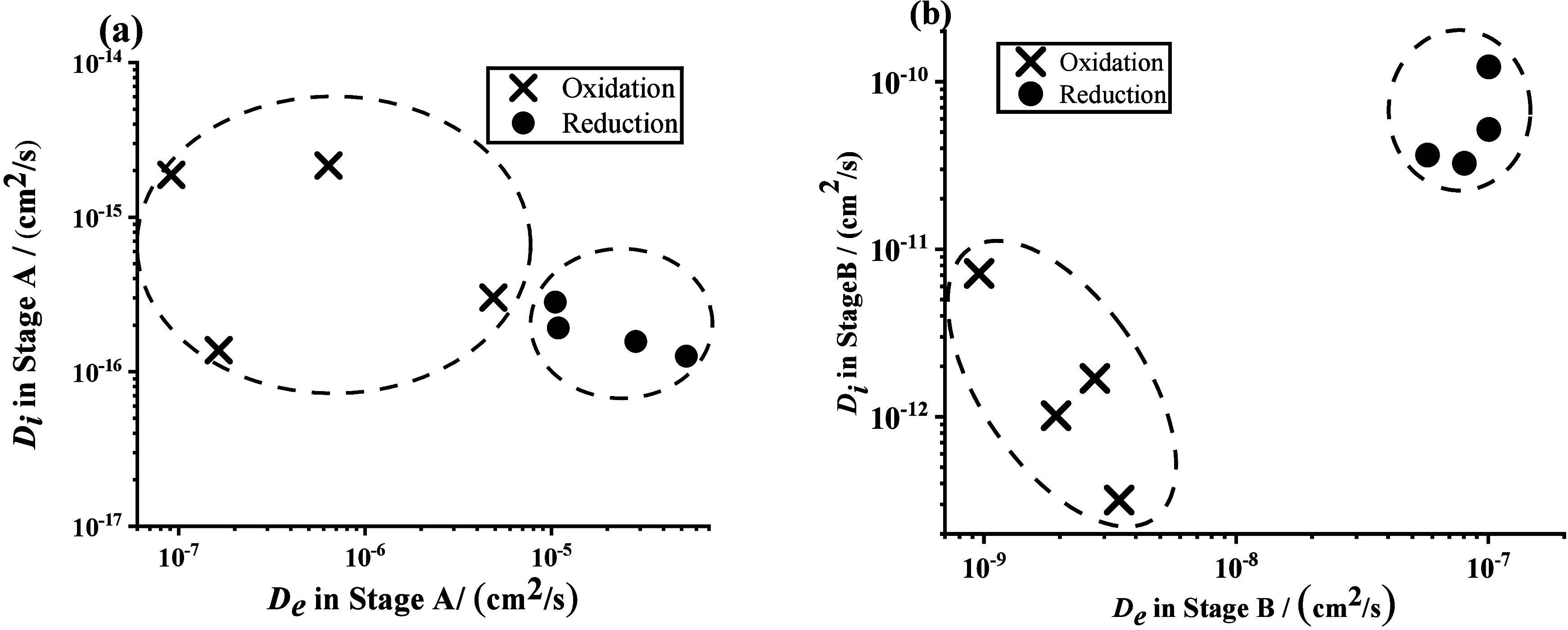
Distribution of *D*_*i*_ and *D*_*e*_ in (a) stage A and (b) stage B. Black crosses (**×**) and black circles (●) represent the data points of the oxidation and reduction on independently measured MOF samples, respectively.

**Table 1:** *D*_*e*_ and *D*_*i*_ of Oxidation/Reduction in Stages A and B

	Stage A		Stage B	
Redox Reaction	*D*_*e*_/(cm^2^/s)	*D*_*i*_/(cm^2^/s)	*D*_*e*_/(cm^2^/s)	*D*_*i*_/(cm^2^/s)
Oxidation [Ru^II^(bpy)_2_(bpy-COOH)]^2+/3+^	1.6 × 10^–7^	1.4 × 10^–16^	9.6 × 10^–10^	7.2 × 10^–12^
	4.9 × 10^–6^	3.0 × 10^–16^	2.8 × 10^–9^	1.7 × 10^–12^
	6.4 × 10^–7^	2.2 × 10^–15^	3.4 × 10^–9^	3.2 × 10^–13^
	9.1 × 10^–8^	1.9 × 10^–15^	1.9 × 10^–9^	1.0 × 10^–12^
Average	(3 ± 2) × 10^–7^	(1 ± 1) × 10^–16^	(2 ± 1) × 10^–9^	(3 ± 3) × 10^–12^
Reduction [Ru^II^(bpy)_2_(bpy-COOH)]^2+/1+^	1.1 × 10^–5^	1.9 × 10^–16^	1.0 × 10^–7^	5.2 × 10^–11^
	1.1 × 10^–5^	2.8 × 10^–16^	8.0 × 10^–7^	3.3 × 10^–11^
	5.3 × 10^–5^	1.3 × 10^–16^	5.7 × 10^–8^	3.6 × 10^–11^
	2.8 × 10^–5^	1.6 × 10^–17^	1.0 × 10^–7^	1.2 × 10^–10^
Average	(3 ± 2) × 10^–5^	(2 ± 1) × 10^–16^	(8 ± 2) × 10^–8^	(6 ± 4) × 10^–11^

Figure 5a suggests that reduction through the π* orbitals of bpy ligands had a *D*_*e*_ higher on average than the oxidation through t_2g_ orbital ((3 ± 2) × 10^–7^ cm^2^/s vs (3 ± 2) × 10^–5^ cm^2^/s for reduction and oxidation, respectively. Errors are reported as one standard deviation). Previously, the Morris lab observed that *D*_*i*_ in stage A is generally very small as a bath of ions surrounds the redox active molecules sampled before the excitation pulse.^3,4^ Thus, it is not surprising to see that the *D*_*i*_ values are within error of each other (*D*_*i*_, reduction = (1 ± 1) × 10^–16^ cm^2^/s; *D*_*i*_, oxidation = (19 ± 7) × 10^–17^ cm^2^/s).

For stage B, the relevant diffusion coefficients can be determined via eqs 3 and 4,
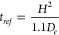
3
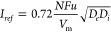
4where *H* is the height of the particle (from the electrode surface to the top of the particles) and *i*_*ref*_ is the value of current at *t*_*ref*_. The calculated *D*_*e*_ and *D*_*i*_ for stage B are listed in Table 1 and visualized in Figure 5.

Bulk redox hopping in Stage B reveals a critical understanding of the function of the interior, which is required to be able to design MOF particles for electrochemical reactions. Figure 5b demonstrates that both *D*_*e*_ and *D*_*i*_ are larger for reduction when compared to oxidation. The average *D*_*e*_ and *D*_*i*_ for the oxidation were (2 ± 1) × 10^–9^ cm^2^/s and (3 ± 3) × 10^–12^ cm^2^/s, respectively, versus (8 ± 2) × 10^–8^ cm^2^/s and (6 ± 4) × 10^–11^ cm^2^/s for reduction.

There are three intriguing observations made from the comparisons of the diffusion coefficients: (1) In both the oxidation and reduction case, *D*_*e*_ of bulk conversion is smaller than surface conversion. (2) The *D*_*e*_ of the reduction process is faster than that of the oxidation process in both surface and bulk regimes of redox hopping. (3) The *D*_*i*_ of the reduction process is higher than that of the oxidation process in bulk regimes of redox hopping (Figure 5). The differences in charge transfer rate were also reflected in the *D*_*app*_ derived from the CA by applying the Cottrell equation, which are, on average, (3.4 ± 1.8) × 10^–12^ cm^2^/s and (3.4 ± 0.5) × 10^–11^ cm^2^/s for oxidation and reduction, respectively.

Redox hopping follows classic Marcus theory; i.e., the rate of redox hopping depends on the self-exchange rate of the redox active species and the distance between redox-hopping centers. Indeed, a positive correlation between the self-exchange rate and spacing between the redox centers has been seen for metallocene-modified NU-1000.^4^ The literature values of self-exchange rates of [Ru^II^(bpy)_3_]^2+^/[Ru^II^(bpy)_2_(bpy^•–^)]^+^ reduction and [Ru^II^(bpy)_3_)]^2+^/[Ru^III^(bpy)_3_)]^3+^ oxidation are 4.2 × 10^8^ M^–1^ s^–1^ and 1.0 × 10^8^ M^–1^ s^–1^, respectively.^41,42^ Thus, the self-exchange rates indicate that, in Ru-NU-1000, the *D*_*e*_ of oxidation should be higher than that of reduction.

To explore any potential difference due to the presence of the carboxylic acid group, density functional theory (DFT) calculations of the inner and outer sphere reorganization energies for oxidation and reduction of [Ru^II^(bpy)_2_(bpy-COOH)] were carried out. Given the ergoneutral nature of the self-exchange reaction, the rate of self-exchange is correlated to the energy barrier associated with reorganization, as there is no thermodynamic driving force. Consistent with the experimental values for the nonsubstituted compound, the DFT reorganization energy was larger for reduction than oxidation, 0.30 eV vs 0.12 eV, respectively (see the Supporting Information). As expected, the calculation shows that the bpy-COOH reorganization dominates the reduction reorganization due to the extra electron. If the charge transport depended on the self-exchange rate, both theory and experiment should indicate that the oxidation has a larger *D*_*e*_ than the reduction process. However, the experimental trend is reversed, suggesting that other factors, like site-to-site hopping distance, play a dominant role in the studied case.

Assuming a uniform distribution of redox centers, the calculated Ru-to-node ratio in Ru-NU-1000 implies there are three [Ru^II^(bpy)_2_(bpy-COOH)]^2+^ centers on nonadjacent node sites in each hexagonal tunnel (Figure 6).^4,43^ Because the hole transport is centered primarily on the metal center (Figure 7), which is proved by the DFT calculation, the distance between adjacent Ru centers for hole transport is approximately 17.3 Å.

**Figure 6:**
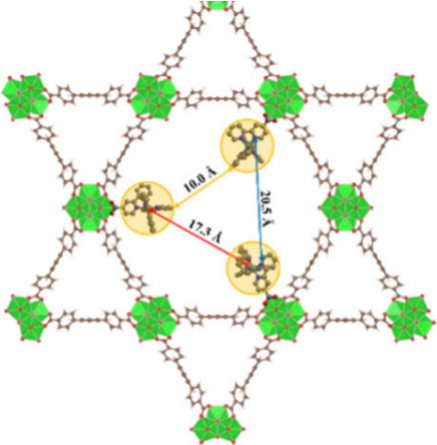
Predicted distribution of [Ru^II^(bpy)_2_(bpy-COOH)]^2+^ centers in the hexagonal tunnel of NU-1000, with arrows showing the estimated distances between (red) Ru centers, (orange) bpy ligands and bpy-COOH ligands (blue).

**Figure 7:**
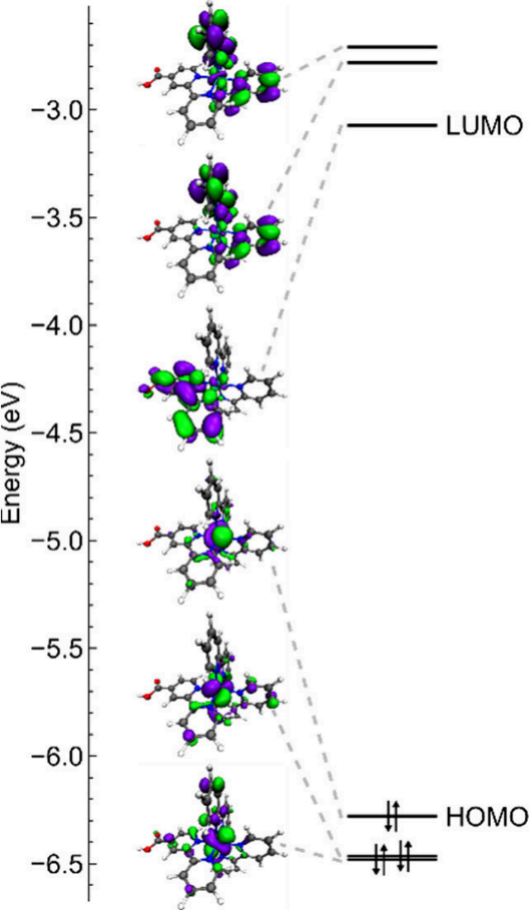
Computational electron configuration of [Ru^II^(bpy)_2_(bpy-COOH)]^2+^ in acetonitrile.

As for reduction, DFT calculations indicate that the first electron transfer primarily occurs to the carboxylate ligand (Figure 7), which matches the result of CV of [Ru^II^(bpy)_2_(bpy-COOH)](PF_6_)_2_ in homogeneous solution (Figure S6). If the electron hopping happens directly from one bpy-COOH ligand to another, the hopping distance would be ∼20.5 Å. Combining the smaller self-exchange rate and the longer hopping distance in the case of reduction, it is impossible to have such an *D*_*e*_ of reduction higher than that of oxidation. So, there must be a shortcut for the electron transfer in the reductive conversion.

Evidence from ultrafast spectroscopic measurements of analogous [Ru^II^(bpy)_3_]^2+^ chromophores indicates that the electron density quickly equilibrates over all present bpy ligands due to the relative similarity in electron affinities.^44,45^ The shared bpy radical character between the excited and reduced states of [Ru^II^(bpy)_3_] means they will likely have similar delocalized electron configurations. In our case, the excitation potential applied for CA is where the first reduction peak ends in the DPV curve (Figure 3b). Due to the inevitable overlap of first and second reduction peaks, the applied potential is negative enough to partially activate the reduction of at least one of the remaining bpy ligands, which could make the electron cloud delocalize across all three bpy ligands. Therefore, we treat the localization of the electron as an electron cloud that encompasses all bpy ligands to calculate a ∼10.0 Å distance between adjacent bpy centers. This distance is significantly smaller than that for the oxidation process (∼17.3 Å). Additionally, the reduced electron cloud is more diffuse compared to the compact density at the oxidized Ru center, which should facilitate good electronic coupling between adjacent bpy centers.

The proposed distances for electron and hole hopping depend on the fact that the molecular motion of the redox sites is restricted through immobilization at the node. Further, molecular restrictions would be amplified in the pore space compared to on the surface of the MOF, which leads to the fact that the *D*_*e*_ is larger in surface conversion than in bulk conversion. This result matches the previous works done by the Morris lab in another MOF redox system.^3,4^

To understand the difference in *D*_*i*_ for reduction and oxidation, we consider the path through which the ions transport and the roles of charge repulsion, ion pairing, and pore crowding. The Scholz model dictates that chemistry originates at the three-phase boundary for both processes. Therefore, the center outermost centers will be converted first.

In the case of oxidation, to balance the additional positive charge that resulted from oxidation, an additional negatively charged ion must diffuse into the MOF from the electrolyte. For the next interior ruthenium center, once oxidized, an ion from the free electrolyte must diffuse through a pore environment that already contains an additional ion (i.e., a more crowded channel), or it must break the ion pairing interactions of the first redox center and through a site-to-site population process move the ions further into the crystallite. In the other case, the reduction process will also originate at the crystal surface, in which the outermost [Ru^II^(bpy)_2_(bpy-COOH)]^2+^ centers will be reduced. Upon reduction, a negative ion deintercalates from the crystallite and enters the free electrolyte. For the inner ruthenium centers, upon reduction, the released ion has a more open channel to transport to the free electrolyte. Additionally, given that all the ruthenium centers in its path are already reduced, charge-balanced, and carry a lower effective charge, these sites would have minimal attraction to hinder ion motion.

Considering that ion diffusion is the rate-determining step in most redox hopping systems, in the case of [Ru^II^(bpy)_2_(bpy-COOH)]^2+^, reduction would be the preferred charge transfer direction, i.e., Ru-NU-1000 would be better at reductive electrocatalysis. Given that many inorganic catalysts for transformations of interest (proton reduction, CO_2_ reduction, water splitting, etc.) feature similar motifs, i.e., positively charged metal centers, we hypothesize that electrocatalysis by MOFs would be more efficient for reductive processes in most cases. The experimental validation of how general this rule is will be the focus of future investigations.

The dependence of reaction type (reduction or oxidation) on redox hopping in MOFs was investigated by the chronoamperometry study of NU-1000 film modified with [Ru^II^(bpy)_2_(bpy-COOH)]^2+^ centers. In both surface conversion and bulk conversion of redox centers, the reductive process, where an electron transfers to the π* orbital on the bpy ligands, exhibited faster ion and electron transport. Indeed, in bulk conversion, the average *D*_*e*_ and *D*_*i*_ for the oxidation were (2 ± 1) × 10^–9^ cm^2^/s and (3 ± 3) × 10^–12^ cm^2^/s, respectively, versus (8 ± 2) × 10^–8^ cm^2^/s and (6 ± 4) × 10^–11^ cm^2^/s for the reduction. Given that the observed trend was at odds with reported (and calculated) self-exchange rates, we posit that the distance between the redox active moieties is dominant in determining the electron hopping rate between redox centers. For ion motion, the transport rates can be understood in terms of steric crowding of the transport channels during oxidation and breaking ion-pairing interactions to enable site-to-site transport. This design rule could be widely applied to many important MOF platforms given the similarities between the [Ru^II^(bpy)_2_(bpy-COOH)]^2+^ centers and common inorganic catalysts.

Building on our discoveries, we are keen to explore redox-active complex species with conjugated ligand structures, which could enhance hole transfer during electrochemical oxidation. Previous studies have shown that one-electron oxidation of nickel(II) porphyrin in specific solvent and electrolyte environments can generate a nickel(II) porphyrin π-radical cation.^46^ Introducing such radical-cation complexes (or those with analogous behavior) into MOFs could yield p-type semiconductor-like properties. This advancement could broaden the electrocatalytic applications of MOFs and enhance their utility in various technological fields.

## Supporting Information Available

Detailed synthetic and experimental procedures, UV–vis spectra, NMR spectra, SEM images, CV, PXRD patterns, detailed electrochemical procedures and results, frontier molecular orbitals, coordinates, and step-by-step sample calculation of the model applied (PDF)
