# Supplementary material for: Reaction-Type-Dependent Behavior of Redox-Hopping in MOFs—Does Charge Transport Have a Preferred Direction?
Source: J Phys Chem Lett. 2024 Nov 21;15(48):11919–26. doi: 10.1021/acs.jpclett.4c01674 (PMC11626502; doi:10.1021/acs.jpclett.4c01674)
Supplement: Supplementary file 1 — jz4c01674_si_001.pdf [file jz4c01674_si_001.pdf]

Reaction-Type-Dependent Behavior of Redox-Hopping in MOFs—Does Charge Transport Have a Preferred Direction?

Minliang Yan[a], Zaya Bowman[b], Zachary J. Knepp[c], Lisa A. Fredin[c], and Amanda J. Morris*[a,d]

[a]Macromolecules Innovation Institute, Virginia Polytechnic Institute and State University, Blacksburg, Virginia 24061, United States.

[b]Department of Chemical Engineering and Department of Chemical Engineering, Virginia Polytechnic Institute and State University, Blacksburg, Virginia 24061, United States.

[c]Department of Chemistry, Lehigh University, Bethlehem, Pennsylvania 18015, United States.

[d]Department of Chemistry, Virginia Polytechnic Institute and State University, Blacksburg, Virginia 24061, United States

*Email: ajmorris@vt.edu

Supporting Information

Synthesis of NU-1000. The preparation of NU-1000 was following a slightly modified procedure from a previous literature.[1] 98 mg of $ZrOCl_2.8H_2O$ and 2 g of benzoic acid were mixed in 8 mL of N, N-dimethylformamide (DMF) in a 6-dram vial. The mixture was sonicated for 15-30 minutes till a uniform and clear solution was formed. The solution was heated in oven at 100 °C for 1 h. After taking it out and cooling down to room temperature, 40 mg of 1, 3, 6, 8-tetrakis(p-benzoate)pyrene (H_4TBAPy) and 40 µL of trifluoroacetic acid (TFA) were added into the mixture and sonicated for 15 min. The yellow mixture was placed in an oven at 100 °C for 18 h. After cooling down to room temperature, the yellow precipitation was isolated by centrifuge and washed with fresh DMF 3 times (soaked for 1 h between washes). The resulting yellow precipitation was suspended in a mixture of 12 mL of DMF and 0.33 mL of 12 M HCl. The mixture was heated in an oven at 100 °C for 18 h. After cooling down to room temperature, the powder was isolated by centrifuge and washed with 12 mL of fresh DMF (soaked for 1 h between washes for the first two washes and soaked overnight for the third time) and 12 mL of acetone (soaked for 1 h between washes for the first two washes and soaked overnight for the third time). After washing, the powder was collected by centrifuge and dried in a vacuum oven at 80 °C overnight.

Synthesis of $[Ru^{II}(bpy)_2(bpy\text{-}COOH)](PF_6)_2$. The preparation of $[Ru^{II}(bpy)_2(bpy\text{-}COOH)](PF_6)_2$ was following a modified procedure from a previous literature[2]. 291.38 mg of $Ru(bpy)_2Cl_2.2H_2O$ and 112 mg of 4-carboxy-2,2'-bypiridine (bpy-COOH) were added into a mixture of 5 mL of methanol and 5 mL of water. The mixture was heated and reflexed overnight to get a dark orange solution. The solution was filtered, and the filtrate was roto-evaporated to nearly dry. 5 mL of DI water with a few drops of concentrated HCl was added into to redissolve the solid, then the solution was roto-evaporated to dry again. The dark-orange solid was collected and suspended in ~3.5 mL of saturated NH_4PF_6 aqueous solution by ultrasonication and stirred overnight to replace Cl^- with PF_6^-. The color of suspending particles turned from dark red to bright orange during the counter ion exchange. The bright-orange precipitation was collected by filtration, washed with cold DI water, and dried in vacuum oven overnight.

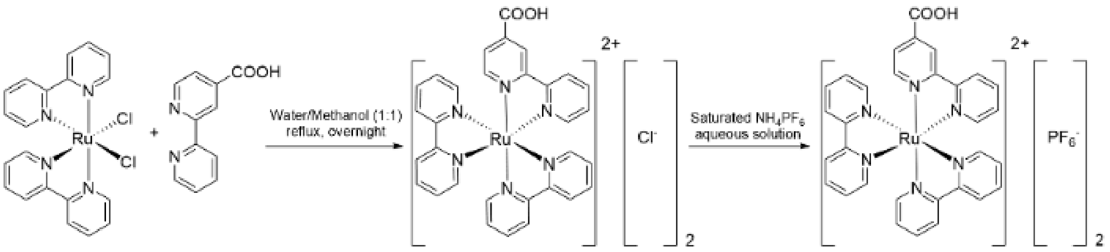

Figure S1. Preparing procedure of [Ru^{II}(bpy)$_2$(bpy-COOH)](PF$_6$)$_2$

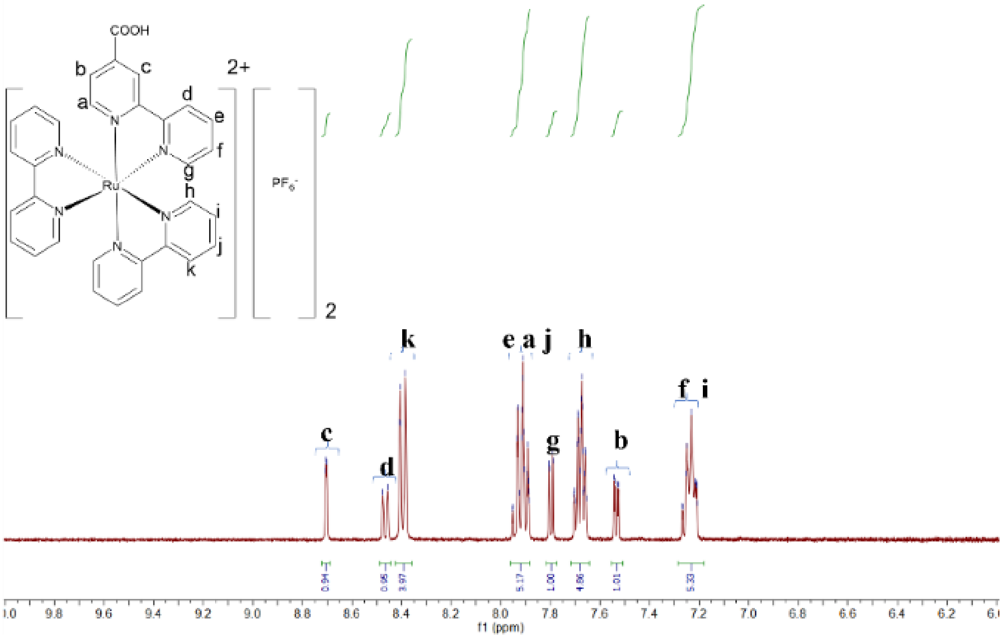

Figure S2. ^{1}H NMR spectrum of [Ru^{II}(bpy)$_2$(bpy-COOH)](PF$_6$)$_2$

Spectroscopy of [Ru^{II}(bpy)$_2$(bpy-COOH)](PF$_6$)$_2$.

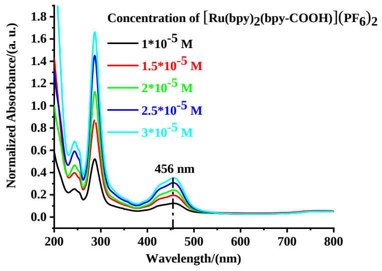

Figure S3. UV-Vis spectroscopy of [Ru^{II}(bpy)$_2$(bpy-COOH)](PF$_6$)$_2$.

Solvent-assistant ligand incorporation (SALI) of NU-1000. The Ru-complex with carboxylic acid group was anchored to NU-1000 following a modified procedure from a previous literature.[3] 30 mg of NU-1000 and 108 mg of [Ru^{II}(bpy)$_2$(bpy-COOH)] (PF$_6$)$_2$ in 1.2 mL of DMF in a 1-dram vial. The mixture was heated in a sand bath at 100 °C for 7 days with continuous stirring at 50 rpm. The mixture was shaken once per day to make the powder stacked on bottom to suspend. After cooling down to room temperature, the precipitation was washed with fresh DMF till the supernatant became colorless (soaked for 1 h between washes) and washed with acetone and diethyl ether for 3 and 2 times, respectively. After washing, the particle was collected by centrifuge and dried in a vacuum oven at 80 °C overnight.

Structural Characterization of NU-1000 and Ru-NU-1000. The SEM images (Fig. S4) before and after the SALI process also indicated that the shape and size of the NU-1000 particles before and after SALI have the same geometry and morphology.

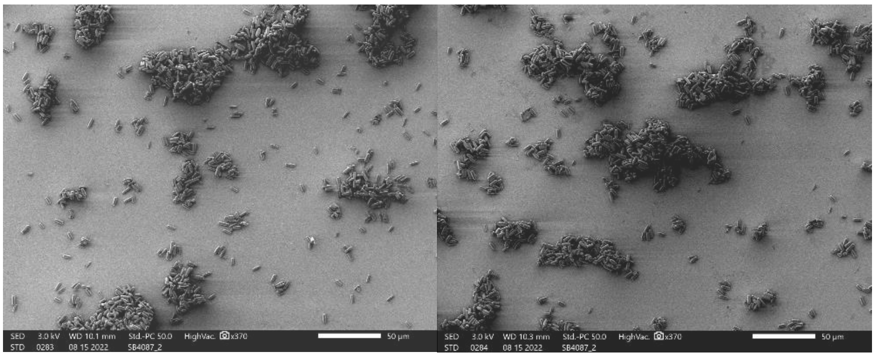

Figure S4. SEM image of NU-1000 before (left) and after (right) the SALI process.

Electrophoretic deposition of Ru-NU-1000

The MOF film was prepared following a modified procedure of previous literature. FTO slides were cleaned by sonication in Alconox solution and DI water for 15 minutes in sequence. After washing, the slides were dried by airflow. 15 mg of Ru-NU-1000 was suspended in 25 mL of toluene in a 6-dram vial by ultrasonication. The solution was stirred at 320 rpm throughout the deposition process. Two FTO slides were 1-cm spaced with the conductive sides facing each other and submerged in the suspension. A constant potential of 135 V was applied between the FTO slides for 3 h to achieve the deposition.

Determination of SALI loading level. The loading level of Ru-NU-1000 was measured by ^{1}H-NMR via the following procedure. ~3 mg of the Ru-NU-1000 with ~5 drops of concentrated H_2SO_4 were added into 600 µL of d6-DMSO. The mixture was heated in a sand bath at 80 °C for 1 h till the particle was fully dissolved. The solution was cooled down to room temperature, well sonicated, and performed by ^{1}H-NMR.

^{1}H NMR was used to determining the ratio of Ru redox center and metal node. Fig. S5 is the ^{1}H NMR spectrum of digested Ru-NU-1000, and the ratio between Ru complex and the organic linker (H4TBAPy) was calculated by the following equation:

$$\frac{\text{Ru complex}}{\text{Linker (H4TBAPy)}} = \frac{\text{Integrated area of selected Ru peak(s)}}{\text{Number of H(s) giving the selected Ru peak(s)}} * \frac{\text{Number of H(s) giving the selected linker peak(s)}}{\text{Integrated area of selected linker peak(s)}} \quad \text{(S1)}$$

Peaks selected to calculate the loading ratio are marked in the NMR spectrum. To guarantee the preciosity, the selected peaks are as isolated as possible to get rid of the overlap with adjacent peaks. Through eq. 1 above, the ratio between Ru complex and linker was calculated to be ~2:1. Considering the structure of NU-1000, the ratio between linkers and oxo-metal nodes is 2:1, the ratio between Ru complex and Zr node is ~1:1, indicating that there is one Ru complex anchored to each Zr node. With the assumption of uniform distribution of Ru humidity and knowing the crystalline structure of NU-1000, the distance between Ru redox centers can be calculated, hence, the hopping distance can be determined.

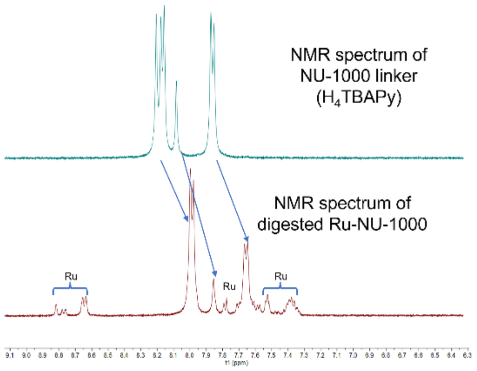

Figure S5. ^{1}H NMR spectrum of H_4TBAPy (top) and digested Ru-NU-1000 (bottom) with the arrows showing corresponding peaks. The difference in chemical shift values is due to the addition of acid.

Electrochemical measurements. All the electrochemical measurements were performed with an Wavenow potentiostat (Pine) using a three-electrode setup. For the cyclic voltammetry of $[Ru^{II}(bpy)_2(bpy\text{-}COOH)](PF_6)_2$ in acetonitrile solution (Fig. S6), a glassy carbon electrode, a platinum wire and a non-aqueous $Ag/AgNO_3$ reference electrode were used as working, counter, and reference electrodes, respectively. 0.1 M tetra(n-butyl) ammonium hexafluorophosphate ($TBAPF_6$) solution in acetonitrile was used as supporting electrolyte.

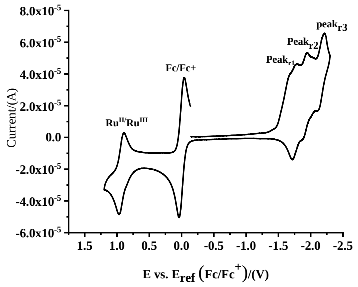

Figure S6. Cyclic voltammetry of $[Ru^{II}(bpy)_2(bpy\text{-}COOH)](PF_6)_2$ in acetonitrile, with $TBAPF_6$ as the supporting electrolyte. Scan rate: 100 mv/s. All potentials are converted from vs. $Ag/AgNO_3$ to vs. Fc/Fc+.

$E_{1/2}$ of $[Ru^{II}(bpy)_2(bpy\text{-}COOH)]^{2+}/[Ru(bpy)_2(bpy\text{-}COOH)]^{3+}$ redox pair was located at 903 mv.

$E_{1/2}$ of reduction peak 1 ($peak_{r1}$) from the $[Ru^{II}(bpy)_2(bpy\text{-}COOH)]^{2+}/[Ru^{II}(bpy)_2(bpy^{\bullet -}\text{-}COOH)]^{+}$ pair was located at -1760 mv.

$E_{1/2}$ of reduction peak 2 ($peak_{r2}$) from the $[Ru^{II}(bpy)_2(bpy^{\bullet -}\text{-}COOH)]^{+}/[Ru^{II}(bpy^{\bullet -})(bpy)(bpy^{\bullet -}\text{-}COOH)]$ pair was located at -1950 mv.

$E_{1/2}$ of reduction peak 3 ($peak_{r3}$) from the $[Ru^{III}(bpy)(bpy^{\bullet -})(bpy^{\bullet -}\text{-}COOH)]/[Ru^{III}(bpy^{\bullet -})_2(bpy^{\bullet -}\text{-}COOH)]^{-}$ pair was located at -2200 mv.

For the electrochemical measurements of MOF film, a FTO slide with Ru-NU-1000 anchored, a platinum mesh and a non-aqueous $Ag/AgNO_3$ reference electrode were used as working, counter, and reference electrodes, respectively. 0.1 M $TBAPF_6$ solution in acetonitrile was used as supporting electrolyte. Prior to the chronoamperometry (CA), differential pulse voltammetry (DPV) of Ru-NU-1000 film was used to determine the excitation potential for chronoamperometry.

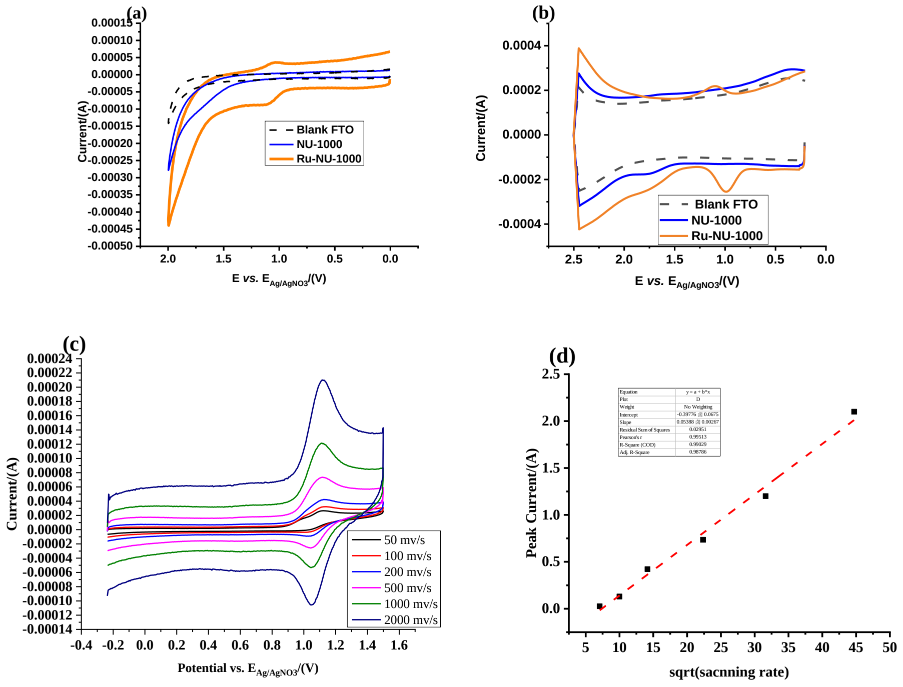

Figure S7. (a) CV of blank FTO, pristine NU-1000, and Ru-NU-1000 in TBAPF6 solution in acetonitrile (0.1 M). Scan rate: 100 mv/s (b) DPV of blank FTO, pristine NU-1000, and Ru-NU-1000 in TBAPF6 solution in acetonitrile (0.1 M). Period = 30 ms, width = 50 ms, height = 50 mv, increment = 5 mv. (c) scan-rate-dependent CV of Ru-NU-1000 of $Ru^{II/III}$ in acetonitrile solution of $TBAPF_6$ (0.1 M) (d) corresponding peak current vs. scan-rate plot on the oxidation side.

CV and DPV of blank FTO, pristine NU-1000, and Ru-NU-1000 in TBAPF6 solution in acetonitrile (0.1 M) were also demonstrated in Fig. S7c and S7d, respectively, to show that the oxidation of NU-1000 will not overlap with the $Ru^{II/III}$ peak.

Scan-rate-dependent CV of $Ru^{II/III}$ was shown in Fig. S7e and the corresponding plot of peak current *vs.* scan rate was included in Fig. S9f. The linear relationship of peak current *vs.* scan rate indicated that the process is diffusion-controlled.

Quantification of D_e and D_i in Ru-NU-1000

The current-time CA responses of Ru-NU-1000 in the reduction and oxidation processes were shown in Fig. 2. According to the Scholz model, the electrons hops along the crystal-solution boundary in a short period at the beginning of conversion, which is treated as stage A. After all the surface redox sites are converted, the electrons and ions need to migrate from the interface into the bulk crystal and this stage is treated as stage B. [4,5] By transforming the CA response to $i\sqrt{t}$, the two stages can be distinguished by the moment when the maximum value of $i\sqrt{t}$ shows up. To determine the t_{ref} of Ru-NU-1000 samples, the examples of $i\sqrt{t}\ vs. \sqrt{t}$ curves of oxidation and reduction were plotted in Fig. 2c and 2d.

In stage A, the relationship between current and time should follow eq. S1

$$i(\mathrm{t}) = \frac{\mathrm{NF}}{\mathrm{V_m}}\left[\frac{1}{1+\exp(-\varphi)}\right]\left[\mathrm{u}\left(\frac{\Delta\mathrm{x}\sqrt{D_e}+\Delta\mathrm{z}\sqrt{D_i}}{2\sqrt{\pi\mathrm{t}}}+\sqrt{D_e D_i}\right)-4D_i\sqrt{2D_e\mathrm{t}}\right] \quad \text{(S2)}$$

Where N is the number of particles in the MOF film, F is Faraday's constant, V_m is the molar volume of NU-1000, u is the length of three-phase junction (the parameter of particle-electrode interface), D_e is the electron hopping coefficient, D_i is the ion diffusion coefficient, Δx_0 is the ion hopping distance, and Δz_0 is the electron hopping distance. φ is defined as:

$$\varphi = F/RT(E - E_f) \quad (S3)$$

Where F is Faraday's constant, R is gas constant, T is the temperature, E is the applied excitation potential in chronoamperometry, E_f is the formal potential of the redox pair (the redox pair is $[Ru^{II}(bpy)_2(bpy\text{-}COOH)]^{2+}/[Ru^{III}(bpy)_2(bpy\text{-}COOH)]^{3+}$ for oxidation and $[Ru^{II}(bpy)_2(bpy^{\bullet -}\text{-}COOH)]^{1+}/ [Ru^{II}(bpy)_2(bpy\text{-}COOH)]^{2+}$ for reduction, respectively). φ was calculated and used respectively for each MOF film.

Based on the previous literature, the molar volume of NU-1000 is calculated by $V_m = \frac{molar\ mass}{density} = \frac{2180\ g/mol}{0.473\ g/cm^3} = 4609.598\ cm^3/mol$, the electron hopping distance (Δz_0) and ion diffusion distance (Δx_0) are 20.839 Å and 17.389 Å, respectively[5].

u is determined by the SEM image of Ru-NU-1000: considering the hexagonal cross-section of NU-1000 particles, the parameter of particle-electrode interface is given by $u = 2 * (l + b) = 2 * l + 2 * \frac{w}{2} = 2 * l + w$. The height of Ru-NU-1000 particles was calculated in a similar (Fig. S8). The dimension of MOF particle was calculated and used respectively for batch of Ru-NU-1000.

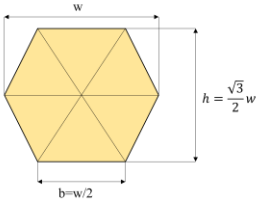

Figure S8. Cross-section schematic of Ru-NU-1000, illustrating the relationship between the width on crystal-electrode interface (b) and the width measured by SEM (w).

Based on a previous literature, N is calculated by the charge, Q, passing through the circuit during the reaction. The total number of Ru center in one MOF film, n, is Q/F, where F is the Faraday's constant.[5] Based on the previous loading ratio calculation, the ratio between Ru center and Zr node is ~1:1, therefore, the number of Ru centers in a single MOF particle can be calculated by $\frac{volume\ of\ each\ MOF\ particle}{molar\ volume}$. The number of MOF particles in a film, N, is given by $N = \frac{n}{number\ of\ Ru\ centers\ in\ each\ MOF\ particle}$. The number of MOF particle was calculated and used respectively for each MOF film.

SEM Scanning electron microscopy (SEM) image was performed at a JEOL IT500 scanning electron microscope.

PXRD Powder X-ray diffraction (PXRD) pattern was collected with Rigaku Miniflex instrument, operating at 2 mA and 40 KV using copper radiation (Cu Kα, λ=1.5418 Å).

Density Functional Theory

$[Ru^{II}(bpy)_2(bpy^{\bullet -}\text{-}COOH)]^{1+}$, $[Ru^{II}(bpy)_2(bpy\text{-}COOH)]^{2+}$, and $[Ru^{III}(bpy)_2(bpy\text{-}COOH)]^{3+}$ were optimized with and without a MeCN polarizable continuum model (PCM) at the UB3LYP-D3BJ/6-311G(d,p)[H,C,N,O]/SDD[Ru] level of theory with the Gaussian16 program using default convergence criteria[6]. Structures were confirmed to be optimized through the lack of imaginary modes in the predicted vibrational frequencies. Root mean square deviations (RMSD) were calculated with respect to the optimized $[Ru^{II}(bpy)_2(bpy\text{-}COOH)]^{2+}$ species for the optimized reduced $[Ru^{II}(bpy)_2(bpy^{\bullet -}\text{-}COOH)]^{1+}$ and oxidized $[Ru^{III}(bpy)2(bpy\text{-}COOH)]^{3+}$ species with and without a MeCN PCM (Table S1). Intramolecular reorganization energies were calculated using the standard four-point method that utilizes the adiabatic potential energy surfaces of an optimized $[Ru^{II}$ (bpy)2(bpy-COOH)] with and without a MeCN PCM (Table S2). Frontier molecular orbitals diagrams were generated for $[Ru^{II}(bpy)_2(bpy^{\bullet -}\text{-}COOH)]^{1+}$, $[Ru^{II}(bpy)_2(bpy\text{-}COOH)]^{2+}$, and $[Ru^{III}(bpy)_2(bpy\text{-}COOH)]^{3+}$ in MeCN PCM using VMD software with an isosurface cutoff of 0.02

(Figures S9 – S11)[7].

Table S1: Root mean square deviations of the geometrically optimized structures with respect to the optimized $[Ru^{II}(bpy)_2(bpy\text{-}COOH)]^{2+}$. Charges 1+, 2+, and 3+ refer to the structures $[Ru^{II}(bpy)_2(bpy^{\bullet-}\text{-}COOH)]^{1+}$, $[Ru^{II}(bpy)_2(bpy\text{-}COOH)]^{2+}$, and $[Ru^{III}(bpy)_2(bpy\text{-}COOH)]^{3+}$, respectively.

**Charge**	**RMSD w/ NO PCM (□)**	**RMSD w/ MeCN PCM (□)**
2+ to 1+	0.1689	0.1037
2+ to 3+	0.1053	0.1442

Table S2: Intramolecular reorganization energies (eV). Energies are calculated in (left) and out (right) of the MeCN PCM model. Charges 1+, 2+, and 3+ refer to the structures $[Ru^{II}(bpy)_2(bpy^{\bullet-}\text{-}COOH)]^{1+}$, $[Ru^{II}(bpy)_2(bpy\text{-}COOH)]^{2+}$, and $[Ru^{III}(bpy)_2(bpy\text{-}COOH)]^{3+}$, respectively.

**Charge**	**λ (eV) w/ NO PCM**	**λ (eV) w/ MeCN PCM**
2+ to 1+	0.1858	0.2983
2+ to 3+	0.0925	0.1159

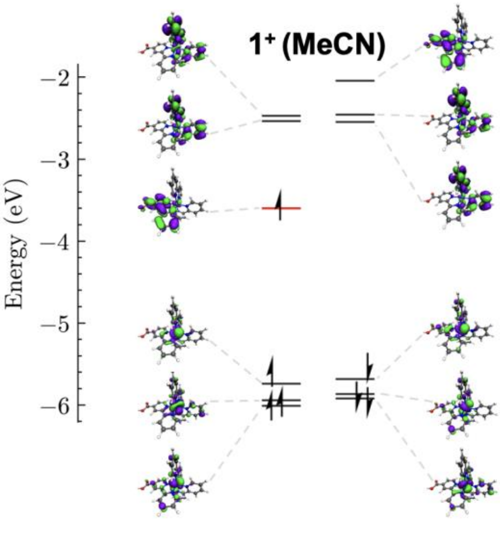

Figure S9: $[Ru^{II}(bpy)_2(bpy^{\bullet-}\text{-}COOH)]^{1+}$ frontier molecular orbital diagram in MeCN PCM at UB3LYP-D3BJ/6-311G(d,p)[H,C,N,O]/SDD[Ru] level of theory. Alpha electrons are on the left and beta electrons are on the right.

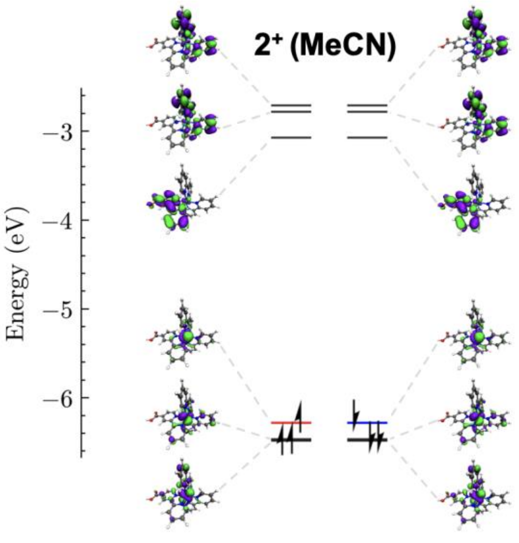

Figure S10: [Ru^{II}(bpy)$_2$(bpy-COOH)]$^{2+}$ frontier molecular orbital diagram in MeCN PCM at UB3LYP-D3BJ/6-311G(d,p)[H,C,N,O]/SDD[Ru] level of theory. Alpha electrons are on the left and beta electrons are on the right.

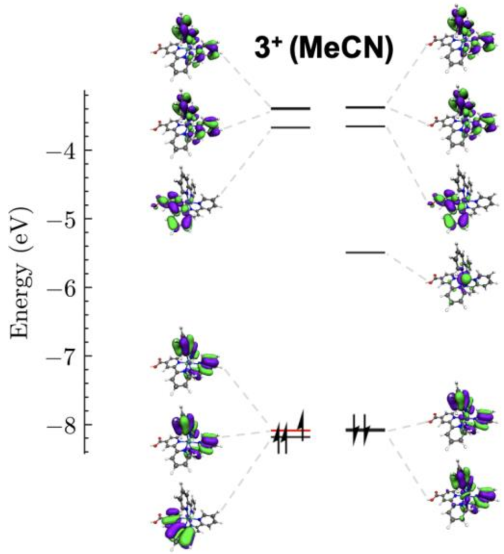

Figure S11: [Ru^{III}(bpy)$_2$(bpy-COOH)]$^{3+}$ frontier molecular orbital diagram in MeCN PCM at UB3LYP-D3BJ/6-311G(d,p)[H,C,N,O]/SDD[Ru] level of theory. Alpha electrons are on the left and beta electrons are on the right.

Table S3: Optimized coordinates of [Ru^{II}(bpy)$_2$(bpy$^{\bullet-}$-COOH)]$^{1+}$ at UB3LYP-D3BJ/6-311G(d,p)[H,C,N,O]/SDD[Ru] level of theory.

C	3.29484583	-2.6034759	1.98459649
C	2.40801241	-1.9097036	2.81365731
C	1.3675052	-1.2060506	2.23485954
N	1.17294446	-1.1594913	0.90380229
C	2.03922089	-1.8247604	0.080735
C	3.10548841	-2.5581567	0.61504439
C	1.74435107	-1.7239142	-1.3439585
N	0.62609624	-1.0012828	-1.6583629
C	0.25532232	-0.8993187	-2.9481962
C	0.97272929	-1.4729439	-3.9816913
C	2.13517703	-2.1884358	-3.6766029
C	2.51616245	-2.3132114	-2.3527314
C	2.31943445	3.93764334	0.24967955
C	2.54362212	3.0650141	-0.8276535
C	1.78948257	1.91214647	-0.9086681
N	0.84804873	1.58614731	-0.0017025
C	0.62848461	2.42059136	1.06708483
C	1.3656027	3.61230597	1.19017307
C	-0.3905821	1.98623438	1.99532145
N	-1.0346013	0.8092198	1.66531507
C	-2.017985	0.35692816	2.47764012
C	-2.3938466	0.99726851	3.63266197
C	-0.7296651	2.67312312	3.16572465

C	-4.0831095	1.70826803	-2.5504465
C	-2.9408164	2.49563634	-2.3752084
C	-1.8751528	1.97498627	-1.6646834
N	-1.8902887	0.7388291	-1.1313393
C	-3.008142	-0.0351624	-1.2810726
C	-4.111569	0.43970803	-1.9994042
C	-2.9375311	-1.3527709	-0.6597391
N	-1.7533071	-1.6403902	-0.0395529
C	-1.5922159	-2.8463471	0.53543943
C	-2.5850971	-3.8087822	0.54686458
C	-3.8094255	-3.518514	-0.0625721
C	-3.9803919	-2.2862389	-0.6680072
Ru	-0.3455019	-0.1144378	-0.048381
H	4.11760917	-3.1678905	2.40445743
H	2.52239569	-1.9097825	3.8892391
H	0.65535813	-0.6544844	2.83250712
H	3.77886883	-3.0907062	-0.0417643
H	-0.6421346	-0.3254896	-3.134553
H	0.63107695	-1.3587186	-5.0016224
H	2.72790695	-2.6408193	-4.4613067
H	3.40950167	-2.8655482	-2.0971637
H	2.8869145	4.8549311	0.34266466
H	3.28753731	3.27757223	-1.5832106
H	1.92060329	1.21113895	-1.7217544
H	1.17903082	4.27414846	2.02432708
H	-2.4977081	-0.5607501	2.1662293
H	-3.1836356	0.60849545	4.25945728
H	-0.2163703	3.58507299	3.43284274
H	-4.933624	2.08308536	-3.1052348
H	-2.8784956	3.49753481	-2.7781468
H	-0.9705876	2.54432136	-1.5028008
H	-4.9856675	-0.1841184	-2.1230307
H	-0.6327931	-3.0191268	1.00325508
H	-2.4042555	-4.7619381	1.02519515
H	-4.6125718	-4.2442247	-0.0630931
H	-4.9197893	-2.043626	-1.1445329
C	-2.1219137	2.87417176	5.24788013
O	-2.985525	2.4779987	5.99277101
O	-1.4093887	4.00367777	5.47716103
H	-1.7392623	4.37648817	6.30771722
C	-1.725904	2.19041633	3.9967462

Table S4: Optimized coordinates of $[Ru^{II}(bpy)_2(bpy\text{-}COOH)]^{2+}$ at UB3LYP-D3BJ/6-311G(d,p)[H,C,N,O]/SDD[Ru] level of theory.

C	3.39174933	-2.6070335	1.89676167
C	2.56328564	-1.8836373	2.74951901
C	1.50223092	-1.175389	2.20728388
N	1.23892395	-1.1625625	0.88888021
C	2.04424926	-1.8645179	0.04565282
C	3.12713374	-2.594543	0.53353376
C	1.679237	-1.7884741	-1.3790208
N	0.6064653	-1.000057	-1.661501
C	0.20328217	-0.8764766	-2.9381019
C	0.84241817	-1.5175991	-3.9878777
C	1.94211151	-2.3242685	-3.7108113
C	2.36091573	-2.4589523	-2.3936032
C	2.4402866	3.89131902	0.34979204
C	2.65770606	3.02783148	-0.7199314
C	1.86932319	1.89322076	-0.8339299
N	0.89871599	1.59533894	0.04738013
C	0.67971632	2.43192954	1.09814664
C	1.44162299	3.58725242	1.26564348
C	-0.4008642	2.02198144	2.00966633
N	-1.0085676	0.8444531	1.69738173
C	-2.01145	0.39790498	2.47665681
C	-2.4493448	1.08663328	3.59340863
C	-0.7980338	2.76142401	3.12169642
C	-4.148141	1.73660788	-2.3737081
C	-3.0239414	2.53856338	-2.2007572
C	-1.925079	2.01380769	-1.538477
N	-1.9008764	0.75788527	-1.0591858
C	-2.9958642	-0.034198	-1.2204788
C	-4.1304886	0.43969805	-1.8774682
C	-2.8778028	-1.3905586	-0.6590346
N	-1.7029094	-1.6556519	-0.0252972
C	-1.5108373	-2.8714682	0.51545275
C	-2.468476	-3.8722356	0.46060413
C	-3.6747007	-3.6111692	-0.1825493
C	-3.8772794	-2.3584753	-0.7467623
Ru	-0.3114577	-0.1020728	-0.0174925
H	4.23036658	-3.1705194	2.28537745
H	2.73237092	-1.8638668	3.81792977
H	0.83222671	-0.6006992	2.83120952
H	3.7623315	-3.1482146	-0.1430586
H	-0.6541546	-0.2400042	-3.1057883
H	0.48039963	-1.3850176	-4.9987405
H	2.46438062	-2.8411572	-4.5057193

H	3.21094294	-3.0841371	-2.1603588
H	3.03836122	4.78550904	0.47033653
H	3.42555777	3.22451558	-1.4562893
H	2.0021317	1.19477214	-1.6481828
H	1.26034493	4.24420093	2.10443909
H	-2.460342	-0.5401247	2.18277794
H	-3.2565097	0.70802261	4.20649167
H	-0.3186513	3.69630538	3.37091186
H	-5.0247288	2.11416293	-2.8844592
H	-2.9941542	3.55531069	-2.5692793
H	-1.0301973	2.59944445	-1.3811825
H	-4.9964253	-0.1951229	-1.9996677
H	-0.5601522	-3.0283858	1.00531745
H	-2.2671251	-4.8340025	0.91329718
H	-4.444663	-4.3694927	-0.2450843
H	-4.8070095	-2.1400027	-1.2523937
C	-2.3175974	3.0307102	5.13727607
O	-3.2103929	2.62814442	5.83108394
O	-1.6365483	4.17025022	5.33429475
H	-1.9916985	4.60362928	6.12671242
C	-1.8318455	2.29175035	3.92413861

Table S5: Optimized coordinates of $[Ru^{III}(bpy)_2(bpy\text{-}COOH)]^{3+}$ at UB3LYP-D3BJ/6-311G(d,p)[H,C,N,O]/SDD[Ru] level of theory.

C	1.87856395	-3.6907855	-2.7640832
C	0.54769463	-3.2828112	-2.8053404
C	0.14656269	-2.233503	-1.9937282
N	1.00424408	-1.5912058	-1.1790433
C	2.3089711	-1.9829106	-1.1239717
C	2.76331558	-3.0359289	-1.9137126
C	3.15419536	-1.227546	-0.1862418
N	2.5171523	-0.2451393	0.51222781
C	3.20962873	0.51467289	1.38119551
C	4.56283161	0.32301158	1.61100782
C	5.2261464	-0.6802427	0.90898886
C	4.51448603	-1.4581784	0.00173136
C	-1.4281744	-3.4496448	2.94857098
C	-0.0519804	-3.2395482	2.98763972
C	0.49669974	-2.2595065	2.17563868
N	-0.259533	-1.4988753	1.36225155
C	-1.6072455	-1.6974872	1.30926657
C	-2.2095224	-2.6729552	2.09949882
C	-2.3355254	-0.8289039	0.37211744

N	-1.5646335	0.0488362	-0.3298624
C	-2.1419152	0.90098738	-1.1988575
C	-3.507018	0.90841844	-1.4227774
C	-3.7154876	-0.8616442	0.19040449
C	-0.3663552	3.96228502	2.75563737
C	-0.6651921	2.71674462	3.302085
C	-0.4203816	1.58099049	2.54655359
N	0.1072312	1.64471921	1.30987668
C	0.40016944	2.85721326	0.75984517
C	0.16786344	4.03031226	1.47325013
C	0.95176058	2.81386348	-0.6032565
N	1.08425534	1.56758101	-1.1400531
C	1.59798043	1.42254987	-2.3758446
C	1.98671847	2.50870501	-3.1438016
C	1.85172442	3.78839616	-2.6113597
C	1.33285232	3.93902143	-1.3296115
Ru	0.48252657	-0.0130117	0.08988339
H	2.22400649	-4.5097492	-3.3830521
H	-0.1713347	-3.7674338	-3.4527702
H	-0.876128	-1.8830766	-1.9912468
H	3.79698658	-3.3487219	-1.869377
H	2.65348737	1.28197726	1.901441
H	5.08149886	0.95021777	2.32418913
H	6.28420252	-0.8538703	1.06236674
H	5.02103889	-2.235233	-0.5531206
H	-1.8879625	-4.2098808	3.56807064
H	0.58984204	-3.8240225	3.63377442
H	1.55959264	-2.0620743	2.1717494
H	-3.2779576	-2.8308935	2.05545943
H	-1.4825667	1.57825997	-1.723166
H	-3.9585923	1.5933504	-2.1295556
H	-4.3382534	-1.5514798	0.74143099
H	-0.5491259	4.86977862	3.31794748
H	-1.0827958	2.62203928	4.29589495
H	-0.6378893	0.59331571	2.92857355
H	0.39741706	4.99225349	1.03710009
H	1.68471281	0.41104453	-2.7472405
H	2.38703108	2.34988674	-4.1365498
H	2.14865093	4.65845541	-3.1840623
H	1.229691	4.92738333	-0.9044507
C	-5.7972353	0.04131395	-0.9728727
O	-6.287654	0.80557011	-1.7532507
O	-6.436131	-0.8677841	-0.2326292

H	-7.3891737	-0.8235428	-0.4203835
C	-4.3083392	0.01235962	-0.7166936

Table S6: Optimized coordinates of $[Ru^{II}(bpy)_2(bpy^{\bullet-}\text{-COOH})]^{1+}$ at UB3LYP-D3BJ/6-311G(d,p)[H,C,N,O]/SDD[Ru] level of theory in MeCN PCM.

C	3.37302845	-2.5205756	1.96142449
C	2.48892037	-1.8337218	2.78933659
C	1.41801244	-1.1654151	2.21813199
N	1.19937846	-1.158029	0.89166537
C	2.05667903	-1.8259486	0.07280307
C	3.15251337	-2.5147791	0.59126575
C	1.72500586	-1.7667185	-1.358661
N	0.62695843	-1.0239513	-1.6666601
C	0.23760509	-0.9333139	-2.9501307
C	0.91923894	-1.5596147	-3.9809398
C	2.0500111	-2.3134947	-3.6779896
C	2.45286355	-2.4176199	-2.3540643
C	2.3804787	3.89017471	0.30068892
C	2.59985144	3.02396948	-0.7839303
C	1.82273617	1.88753283	-0.8802876
N	0.86551944	1.57503541	0.01438624
C	0.64227511	2.40552596	1.08665445
C	1.40885217	3.57993524	1.22702545
C	-0.3967386	1.98738422	1.99542908
N	-1.0311337	0.78663057	1.67287879
C	-2.0275561	0.34976655	2.48415526
C	-2.4326631	1.01126169	3.61158749
C	-0.768439	2.69857279	3.13250329
C	-4.1328545	1.7833519	-2.381065
C	-2.9807462	2.55183481	-2.2326065
C	-1.8875488	2.00187866	-1.5837274
N	-1.8945206	0.74999784	-1.0926468
C	-3.0157346	-0.0097277	-1.2318229
C	-4.1468689	0.49149142	-1.8751051
C	-2.9227403	-1.3639705	-0.6692557
N	-1.7390712	-1.6566204	-0.064375
C	-1.5590084	-2.8791343	0.46528172
C	-2.5394285	-3.857126	0.42929037
C	-3.7572828	-3.5657942	-0.1801055
C	-3.9468211	-2.3081076	-0.7347818
Ru	-0.3350991	-0.1268057	-0.0497828
H	4.22074481	-3.0513316	2.37474793
H	2.62301736	-1.8093575	3.86230542

H	0.70455524	-0.6152331	2.81455367
H	3.82809263	-3.040194	-0.0680076
H	-0.6413149	-0.3330416	-3.1378791
H	0.56625376	-1.4532662	-4.9977142
H	2.60757811	-2.8143918	-4.4585832
H	3.32379359	-3.0033247	-2.0978925
H	2.96784014	4.79328402	0.41075839
H	3.35335217	3.22844896	-1.5320074
H	1.94772401	1.1914583	-1.6983394
H	1.23204637	4.23793505	2.06623441
H	-2.4949437	-0.5806295	2.18953691
H	-3.2300383	0.61852853	4.2257054
H	-0.2666002	3.62385105	3.3761295
H	-5.0054751	2.18313973	-2.8808433
H	-2.9247889	3.56454508	-2.6082514
H	-0.9732806	2.55967408	-1.4416374
H	-5.0312711	-0.1205239	-1.9774984
H	-0.6006796	-3.0576122	0.93175583
H	-2.3461356	-4.8246338	0.87236497
H	-4.5456811	-4.3057625	-0.2244748
H	-4.8826387	-2.0643791	-1.2164311
C	-2.198285	2.95334393	5.16348987
O	-3.0829119	2.59420496	5.9280528
O	-1.5015908	4.10565016	5.38520745
H	-1.8609558	4.49015598	6.19729485
C	-1.7836014	2.23807812	3.96771373

Table S7: Optimized coordinates of $[Ru^{II}(bpy)_2(bpy\text{-}COOH)]^{2+}$ at UB3LYP-D3BJ/6-311G(d,p)[H,C,N,O]/SDD[Ru] level of theory in MeCN PCM.

C	3.38758326	-2.6060937	1.88553169
C	2.56074237	-1.8875748	2.74294473
C	1.49664593	-1.1793574	2.2075329
N	1.23317341	-1.1629587	0.89025547
C	2.03611278	-1.8605629	0.04243836
C	3.12088207	-2.5908214	0.52321069
C	1.66793428	-1.7787505	-1.3797787
N	0.59589336	-0.9888338	-1.6559458
C	0.19031048	-0.8529287	-2.9294189
C	0.82797086	-1.4882353	-3.9831209
C	1.92594389	-2.298398	-3.7126452
C	2.3477833	-2.443568	-2.3980009
C	2.45319178	3.86973224	0.36455877
C	2.66429605	3.00833532	-0.7071618

C	1.86830301	1.87977721	-0.8261633
N	0.89731741	1.5889475	0.05525526
C	0.68399629	2.42370161	1.10750004
C	1.45281546	3.57225145	1.28008893
C	-0.4002746	2.01775591	2.0139792
N	-1.0112461	0.84284799	1.69937302
C	-2.020047	0.39888435	2.47081548
C	-2.4601366	1.08996749	3.58436477
C	-0.7989944	2.75852611	3.12273829
C	-4.1311946	1.72125732	-2.3942847
C	-3.0134705	2.52840602	-2.2084443
C	-1.9195412	2.0098916	-1.533823
N	-1.8990848	0.75515278	-1.0543996
C	-2.9876972	-0.0418306	-1.2269529
C	-4.1159769	0.42484561	-1.8977763
C	-2.8705758	-1.3949302	-0.6603703
N	-1.7020721	-1.6530612	-0.0139095
C	-1.5100097	-2.8621731	0.53926304
C	-2.4647872	-3.8650826	0.4799184
C	-3.6638574	-3.6120904	-0.1784017
C	-3.8663816	-2.3647579	-0.7532979
Ru	-0.3157043	-0.1003356	-0.0086006
H	4.22766193	-3.1696903	2.26951919
H	2.73016178	-1.8703136	3.81075618
H	0.8270691	-0.6111027	2.83645294
H	3.75295801	-3.1416236	-0.1576971
H	-0.6641142	-0.2129155	-3.0932056
H	0.46398729	-1.3455621	-4.9913035
H	2.44670442	-2.810396	-4.5110792
H	3.19743681	-3.0696457	-2.1686273
H	3.05707035	4.75900327	0.48799133
H	3.43229535	3.1997573	-1.4439029
H	1.99517674	1.18507398	-1.6435616
H	1.27544092	4.22778643	2.1199948
H	-2.4712665	-0.5367714	2.17624202
H	-3.2720838	0.70560735	4.18502709
H	-0.3123212	3.68957024	3.36813341
H	-5.0026258	2.09382445	-2.9164562
H	-2.9812737	3.54458828	-2.5766349
H	-1.028558	2.59819299	-1.3691284
H	-4.9754645	-0.2156216	-2.0304924
H	-0.5660053	-3.0131866	1.04208165
H	-2.2638396	-4.8217159	0.94224404

H	-4.4305465	-4.3728077	-0.2443922
H	-4.7906882	-2.1503948	-1.2693737
C	-2.3166623	3.03704788	5.12243914
O	-3.2172296	2.65580846	5.83113879
O	-1.6333297	4.17288828	5.32441392
H	-1.990286	4.60515416	6.11585481
C	-1.8390336	2.29200006	3.91915482

Table S8: Optimized coordinates of oxidized $[Ru^{III}(bpy)_2(bpy\text{-}COOH)]^{3+}$ at UB3LYP-D3BJ/6-311G(d,p)[H,C,N,O]/SDD[Ru] level of theory in MeCN PCM.

C	1.97809342	-3.6557474	-2.7353969
C	0.64479018	-3.2659365	-2.8021595
C	0.21525111	-2.2178067	-2.0054524
N	1.05259413	-1.567322	-1.1810875
C	2.35862988	-1.9400184	-1.100929
C	2.84195307	-2.98799	-1.8765075
C	3.17234907	-1.1680342	-0.1538827
N	2.50577854	-0.195783	0.52618118
C	3.16142741	0.58499074	1.40120448
C	4.51294787	0.42053312	1.65429511
C	5.20619409	-0.5749974	0.97440487
C	4.53047161	-1.3740193	0.06079126
C	-1.377054	-3.5070756	2.87325547
C	-0.0071365	-3.2746445	2.91704311
C	0.52729692	-2.2727424	2.12340966
N	-0.2428902	-1.5170071	1.32367894
C	-1.5847234	-1.7377318	1.26596029
C	-2.17254	-2.7329816	2.03760095
C	-2.3232199	-0.8653761	0.3448297
N	-1.5661065	0.0292613	-0.3424685
C	-2.1430434	0.88612584	-1.2009076
C	-3.5076459	0.88176889	-1.4254378
C	-3.7009084	-0.9129916	0.16153484
C	-0.5268274	3.92438461	2.73627466
C	-0.8090675	2.67055685	3.26749959
C	-0.5130722	1.54487664	2.51680647
N	0.04582149	1.63447549	1.29861981
C	0.32572184	2.85400834	0.76444035
C	0.04410201	4.01656781	1.47324998
C	0.91896974	2.82933773	-0.5784097
N	1.07967131	1.59143447	-1.1198558
C	1.62543936	1.45426173	-2.3398405
C	2.02682997	2.54982902	-3.0855853

C	1.86798017	3.82208314	-2.5468673
C	1.31113949	3.9626298	-1.2818701
Ru	0.48080406	-0.0055136	0.08056874
H	2.34377146	-4.4723646	-3.3434582
H	-0.0589719	-3.7601475	-3.45676
H	-0.8093823	-1.8770402	-2.0256012
H	3.8790403	-3.2817145	-1.813749
H	2.58182725	1.34248478	1.90725396
H	5.00246993	1.06504985	2.37052986
H	6.26281887	-0.7278969	1.14905592
H	5.05733802	-2.1473526	-0.4776843
H	-1.8244284	-4.2833402	3.47948392
H	0.64653535	-3.8555168	3.55210182
H	1.58488189	-2.0560758	2.12914218
H	-3.2372916	-2.9050913	1.98953813
H	-1.4904182	1.57317991	-1.7182877
H	-3.9515395	1.57503721	-2.125075
H	-4.3071349	-1.6210267	0.70445792
H	-0.7502542	4.82285766	3.29592743
H	-1.2533778	2.55606511	4.24604546
H	-0.7112257	0.5508356	2.88957178
H	0.26275348	4.98341123	1.04526658
H	1.7282526	0.44757371	-2.7161758
H	2.45622352	2.39789646	-4.0656695
H	2.17586431	4.69765102	-3.1027105
H	1.18732791	4.94400576	-0.8491493
C	-5.7772472	-0.0320312	-0.983946
O	-6.3139759	0.72055553	-1.75774
O	-6.4107877	-0.9552563	-0.2539847
H	-7.3592792	-0.9122293	-0.4541915
C	-4.2976866	-0.0324542	-0.7339854

References

(1) Gomez, I. J.; Arnaiz, B.; Cacioppo, M.; Arcudi, F.; Prato, M. Revisiting the Structural Homogeneity of NU-1000, a Zr-Based Metal-Organic Framework. *J. Mater. Chem. B* **2018**, *6* (35).

(2) Zhou, M.; Robertson, G. P.; Roovers, J. Comparative Study of Ruthenium(II) Tris(Bipyridine) Derivatives for Electrochemiluminescence Application. *Inorg. Chem.* **2005**, *44* (23), 8317–8325.

(3) Gibbons, B.; Bartlett, E. C.; Cai, M.; Yang, X.; Johnson, E. M.; Morris, A. J. Defect Level and Particle Size Effects on the Hydrolysis of a Chemical Warfare Agent Simulant by UiO-66. *Inorg. Chem.* **2021**, *60* (21), 16378–16387.

(4) Schröder, U.; Oldham, K. B.; Myland, J. C.; Mahon, P. J.; Scholz, F. Modelling of Solid State Voltammetry of Immobilized Microcrystals Assuming an Initiation of the Electrochemical Reaction at a Three-Phase Junction. *J. Solid State Electrochem.* **2000**, *4* (6), 314–324.

(5) Cai, M.; Loague, Q.; Morris, A. J. Design Rules for Efficient Charge Transfer in Metal-Organic

Framework Films: The Pore Size Effect. *J. Phys. Chem. Lett.* **2020**, *11* (3), 702–709.

(6) Gaussian 16, Revision C.01, Frisch, M. J.; Trucks, G. W.; Schlegel, H. B.; Scuseria, G. E.; Robb, M. A.; Cheeseman, J. R.; Scalmani, G.; Barone, V.; Petersson, G. A.; Nakatsuji, H.; Li, X.; Caricato, M.; Marenich, A. V.; Bloino, J.; Janesko, B. G.; Gomperts, R.; Mennucci, B.; Hratchian, H. P.; Ortiz, J. V.; Izmaylov, A. F.; Sonnenberg, J. L.; Williams-Young, D.; Ding, F.; Lipparini, F.; Egidi, F.; Goings, J.; Peng, B.; Petrone, A.; Henderson, T.; Ranasinghe, D.; Zakrzewski, V. G.; Gao, J.; Rega, N.; Zheng, G.; Liang, W.; Hada, M.; Ehara, M.; Toyota, K.; Fukuda, R.; Hasegawa, J.; Ishida, M.; Nakajima, T.; Honda, Y.; Kitao, O.; Nakai, H.; Vreven, T.; Throssell, K.; Montgomery, J. A., Jr.; Peralta, J. E.; Ogliaro, F.; Bearpark, M. J.; Heyd, J. J.; Brothers, E. N.; Kudin, K. N.; Staroverov, V. N.; Keith, T. A.; Kobayashi, R.; Normand, J.; Raghavachari, K.; Rendell, A. P.; Burant, J. C.; Iyengar, S. S.; Tomasi, J.; Cossi, M.; Millam, J. M.; Klene, M.; Adamo, C.; Cammi, R.; Ochterski, J. W.; Martin, R. L.; Morokuma, K.; Farkas, O.; Foresman, J. B.; Fox, D. J. Gaussian, Inc., Wallingford CT, 2016.

(7) Humphrey, W., Dalke, A. and Schulten, K., "VMD - Visual Molecular Dynamics", *J. Molec. Graphics*, **1996**, vol. 14, pp. 33-38.
